# Rétinoschisis maculaire bilatéral associé à un rétinoschisis périphérique unilatéral

**DOI:** 10.11604/pamj.2017.28.38.12093

**Published:** 2017-09-14

**Authors:** Hanane Oummad, Maryama Elkaddoumi, Josiane Maré, Mounir Lezrek, Ouafae Cherkaoui

**Affiliations:** 1 Hôpital des Spécialités, Université Mohammed V, Rabat, Maroc

**Keywords:** Décollement de rétine, schisis périphérique, rétinoschisis maculaire, Retinal detachment, peripheral schisis, macular retinoschisis

## Abstract

Le retinoschisis juvénile lié au chromosome X est une affection héréditaire qui affecte habituellement les garçons avec de rare cas d’atteinte du sexe féminin. Les premières manifestations cliniques apparaissent généralement au cours de la première décennie. Il est responsable d’une baisse d’acuité visuelle d’importance variable et lentement progressive. Cette évolution peut être émaillée d’hémorragies dans le vitré et de décollements de rétine volontiers récidivants. Nous présentons le cas d’un patient âgé de 17ans. Au fond d'œil on retrouve remaniement maculaire microkystique stellaire bilatéral, centré sur la fovéola, associé à des schisis périphériques avec décollement de rétine et déchirure des feuillets interne et externe unilatéral.

## Abstract (en)

X-linked juvenile retinoschisis is a hereditary disorder which usually occurs in boys rather than in girls, who are rarely affected. First clinical manifestations usually appear during the first decade. It is responsible for variable severity and slowly progressive vision loss. This progression can be characterized by vitreous hemorrhages and recurrent retinal detachments. We report the case of a 17-year old patient with stellar bilateral microcistic macular rearrangement of the eye-ground, centered on the foveola, associated with peripheral schisis with retinal detachment and unilateral tearing of internal and external layers.

## Introduction

Le retinoschisis juvénile lié au chromosome X est la plus fréquente des dégénérescences maculaires juvéniles qui affecte habituellement les garçons avec de rare cas d’atteinte du sexe féminin [1]. Les premières manifestations cliniques apparaissent généralement au cours de la première décennie. Il est responsable d’une baisse d’acuité visuelle d’importance variable et lentement progressive. Cette évolution peut être émaillée d’hémorragies dans le vitré et de décollements de rétine volontiers récidivants [2].

## Patient et observation

**Figure 1:**
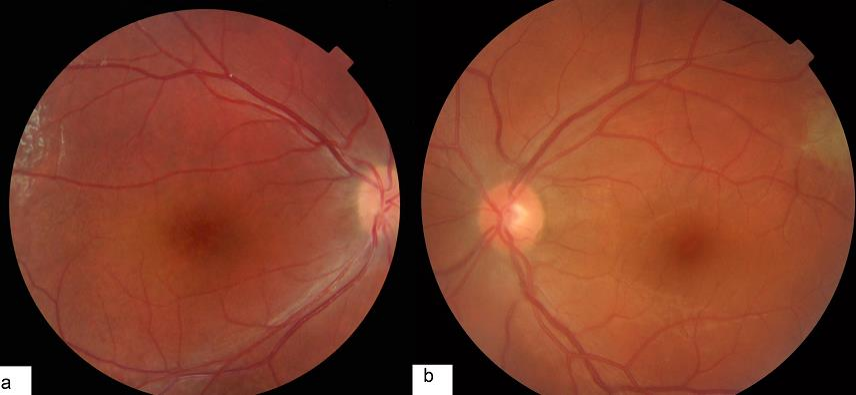
Remaniement maculaire microkystique stellaire bilatéral centré sur la fovéola

**Figure 2:**
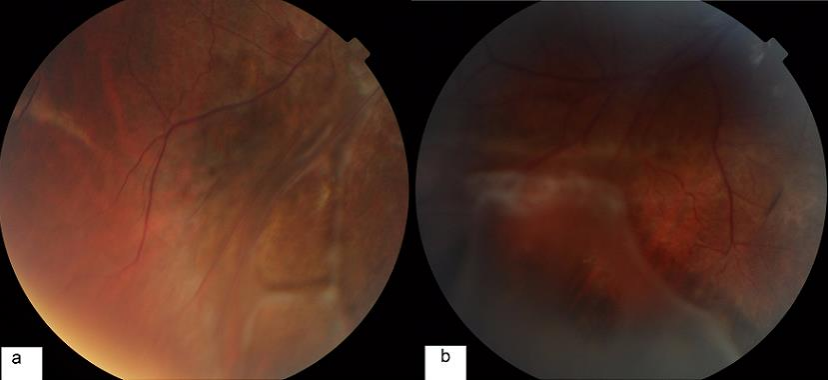
Schisis périphériques avec décollement de rétine et déchirure des feuillets interne et externe de l’œil droit

**Figure 3:**
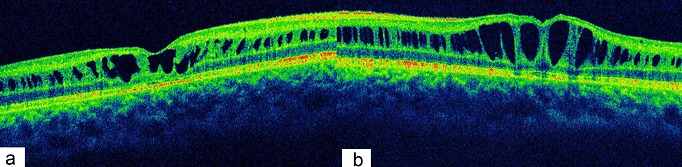
Clivage dans les couches les plus externes de la rétine et formation de logettes kystiques de diamètre décroissant du centre fovéolaire vers la périphérie au niveau de l’oeil droit (a) et l’oeil gauche (b)

Nous présentons le cas d’un patient âgé de 17ans, de sexe masculin. ayant comme antécédent un oncle malvoyant et qui présente une baisse d’acuité visuelle progressive depuis l’enfance non améliorable par le port de correction optique. L’examen ophtalmologique trouve une acuité visuelle à 1/10 en OD et 2/10 en OG inaméliorable. Le segment antérieur est normal en ODG. Au fond d´œil on retrouve remaniement maculaire microkystique stellaire bilatéral, centré sur la fovéola, associé à des schisis périphériques avec décollement de rétine et déchirure des feuillets interne et externe de l’œil droit (Figure 1 et Figure 2). L’OCT objective un clivage dans les couches les plus externes de la rétine et la formation de logettes kystiques de diamètre décroissant du centre fovéolaire vers la périphérie (Figure 3). Une surveillance rétinienne régulière s´impose vu que le décollement de rétine n‘est pas évolutive.

## Discussion

Le rétinoschisis juvénile lié à l’X est une dystrophie héréditaire rare de transmission récessive liée à l’X. Il touche le sexe masculin avec une expressivité variable, le gène connu en cause étant le XLRS1, se situant en position Xp22.2-p22.1 [3]. IL résulte d´un clivage au sein de la couche des fibres nerveuses secondaire à la dégénérescence des cellules de Müller [4]. Le diagnostic se fait souvent à l´âge scolaire devant une baisse de l´acuité visuelle. Le rétinoschisis maculaire est le signe pathognomique [1]. Le rétinoschisis périphérique est rencontré dans 50 % des cas en temporal inférieur [1, 5]. C´est une pathologie évolutive et de pronostic sévère. La surveillance doit être étroite pour dépister et prendre en charge les complications (décollement de rétine ou hémorragie du vitré) secondaire à des anomalies localisées au niveau des couches internes de la rétine [4]. Le décollement de la rétine sur rétinoschisis est une pathologie rare et ils sont le plus souvent localisés. Le traitement chirurgical ne peut être indiqué que dans les formes évolutives du décollement. Dans ces cas une chirurgie par voie endo-oculaire est indiquée permettant une bonne réapplication de la rétine, suivie d’une photocoagulation au laser des déhiscences du feuillet externe [5, 6].

## Conclusion

Le schisis juvénile lié au sexe est une pathologie rare qui touche le jeune garçon, sa présentation clinique est variable. Son pronostic reste sombre. L'évolution peut être émaillée de complications graves de la périphérie rétinienne qu'il faut savoir reconnaître précocement et dont le traitement peut être difficile. Le diagnostic d’un schisis localisé sans décollement rétinien constitue une étape importante dans le traitement préventif de ces lésions.

## Conflits d’intérêts

Les auteurs ne déclarent aucun conflit d'intérêts.
